# Iron Deficiency Impairs Dendritic Cell Development and Function, Compromising Host Anti‐Infection Capacity

**DOI:** 10.1002/advs.202408348

**Published:** 2025-04-30

**Authors:** Quanzhong Ren, Xiaotong Xu, Zheng Dong, Jiahuang Qiu, Qing'e Shan, Rui Chen, Yajun Liu, Juan Ma, Sijin Liu

**Affiliations:** ^1^ State Key Laboratory of Environmental Chemistry and Ecotoxicology Research Center for Eco‐Environmental Sciences Chinese Academy of Sciences Beijing 100085 P. R. China; ^2^ JST sarcopenia Research Centre National Center for Orthopaedics Beijing Research Institute of Traumatology and Orthopaedics Beijing Jishuitan Hospital Capital Medical University Beijing 100035 P. R. China; ^3^ Department of Toxicology and Sanitary Chemistry School of Public Health Capital Medical University Beijing 100069 P. R. China; ^4^ University of Chinese Academy of Sciences Beijing 100049 P. R. China; ^5^ Medical Science and Technology Innovation Center Shandong First Medical University & Shandong Academy of Medical Sciences Jinan Shandong 250117 P. R. China

**Keywords:** cellular function, dendritic cells, influenza virus infection, iron deficiency, monocyte–dendritic cell progenitors

## Abstract

The prevalence of acute lower respiratory infections in individuals with iron deficiency (ID) has significantly increased, and is correlated with reduced numbers of immune cells and impaired immune function. Dendritic cells (DCs) play a crucial role in combating the influenza A virus (IAV) by initiating adaptive immune responses. However, the impact of ID on DCs and their response to IAV infection remain unclear. This study showed that ID impairs the antigen‐presenting ability of DCs, thereby hindering their capacity to mediate T‐cell proliferation and clear viruses. The restrictive effects of ID on DCs begin in the bone marrow and specifically affect the monocyte DC progenitor (MDP) stage. A reduction in the number of MDPs and compromised immune potential lead to a decrease in the population and functionality of DCs in the subsequent common DC precursor (CDP) stage in the blood, spleen, and lungs. This study highlights the previously unrecognized impact of ID on DCs and provides valuable insights into immune cell responses and the application of iron supplementation in the fight against viral infections.

## Graphical abstract

Iron deficiency reduces monocyte dendritic cell progenitor (MDP) numbers by inhibiting migration and proliferation while promoting apoptosis, subsequently reducing downstream common dendritic cell precursors (CDP) and dendritic cell (DC). Impaired DCs enter the lungs via blood circulation, where reduced antigen presentation impairs T‐cell activation and antiviral immunity, enhancing susceptibility to infections.

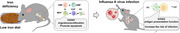

## 1 Introduction

Iron is an essential micronutrient crucial for various physiological functions. An increasing number of studies have demonstrated that anemia ranks as the fifth leading cause of global disease burden and is emerging as a significant public health challenge worldwide.^[^ ^1^ ^]^ More than 50% of anemia cases are caused by iron deficiency (ID).^[^ ^2^ ^]^ ID disturbs the quantity and functionality of immune cells and consequently leads to an elevated prevalence of acute lower respiratory tract infections.^[^ ^3^, ^4^, ^5^, ^6^ ^]^ For example, ID diminishes the neutrophil count and impairs phagocytic capacity and cytokine production.^[^ ^7^, ^8^ ^]^ Alterations in iron levels influence the polarization of macrophages, as evidenced by the fact that excess iron tends to promote the polarization of pro‐inflammatory M1 macrophages, whereas ID tends to favor the polarization of anti‐inflammatory M2 macrophages.^[^ ^9^ ^]^ Furthermore, ID reduces the proportion and maturation of T lymphocytes.^[^ ^10^, ^11^ ^]^ In addition, TH2 cells have larger chelatable iron storage pools, but the iron in these pools is in a relatively inactive and less chelatable state, rendering it less sensitive to the effects of ID compared that in TH1 cells. Therefore, TH2 cells exhibit greater stability in iron‐deficient environments.^[^ ^12^ ^]^ Dendritic cells (DCs) are antigen‐presenting cells. Among non‐professional antigen‐presenting cells such as monocytes and macrophages, only DCs can activate naive T cells, making them critical players in the clearance of viruses and bacteria.^[^ ^13^ ^]^ However, limited in vitro data regarding the impact of ID on DCs have demonstrated that DC maturation is dependent on iron.^[^ ^14^, ^15^ ^]^ The molecular mechanisms underlying the impact of ID on DC function and susceptibility to infection with influenza virus remain inadequately elucidated.^[^ ^14^, ^15^ ^]^

During the outbreaks of coronavirus disease 2019 (COVID‐19), ID individuals were more prone to infection and experienced more severe symptoms than other populations.^[^ ^16^, ^17^, ^18^ ^]^ Existing research indicates that serum iron levels decrease in 90% of COVID‐19 patients. The disease severity and mortality rate were negatively correlated with serum iron levels, indicating that ID may be a significant risk factor for mortality in COVID‐19 patients.^[^ ^16^, ^17^, ^18^ ^]^ Similarly, the influenza virus, which is responsible for 13% of acute lower respiratory tract infections, causes influenza both globally and annually.^[^ ^19^ ^]^ Thus, it is imperative to delve deeper into the impact of ID on influenza viral infections. The innate immune response, followed by an adaptive immune response, is important for viral clearance. A pivotal step in this mechanism involves the recognition of the influenza virus by DCs, which subsequently present antigens to T lymphocytes, thereby facilitating the proliferation and activation of T cells.^[^ ^20^ ^]^ The expansion and activation of virus‐specific T cells are indispensable for the eradication of influenza viruses.^[^ ^21^ ^]^ Impaired T cell function can lead to viral proliferation in the lungs, subsequently triggering excessive infiltration of immune cells from the circulating blood, causing substantial damage to lung tissues and even acute respiratory distress syndrome.^[^ ^22^ ^]^ However, the impact of ID on the response of DCs to influenza virus has not been documented.

Here, we report that ID induces DC development and anti‐viral functions. A direct correlation between the impairment of DC function and reduced antiviral efficacy in iron‐deficient mice was uncovered. By constructing a mouse model of ID and performing human influenza virus A/Puerto Rico/8/34 (H1N1 PR8) infection, we found that the adverse impact of ID on DC function originates in the bone marrow, especially in the monocyte DC progenitor (MDP) stage, which is the stage most affected by ID. Systemic ID leads to a decrease in the abundance of MDPs in the bone marrow and compromises immune‐related functions, which serves as the underlying cause for the diminished antiviral capacity of DCs in the lungs. Our study offers a novel rationale for the management of influenza in individuals with ID.

## 2 Results and Discussion

### 2.1 ID Exacerbates Viral Lung Disease

To ascertain the impact of ID on immune cell protection against viral infection, we began our investigation by assessing the infection severity in mice with ID (“ID mice”) upon infection with H1N1 PR8, a mouse‐adapted human influenza A virus (IAV) typically used in experimental studies.^[^ ^23^ ^]^ We first developed a low‐iron diet‐driven ID mouse model to study systemic disorders of iron homeostasis (Figure S1a,b, Supporting Information), as described.^[^ ^24^, ^25^ ^]^ After 4‐week feeding with low‐iron diet (5.93 ppm), ID mice showed no significant changes in body weight compared with iron‐normal (IN) mice fed with a diet containing 105.26 ppm iron (Figure S1c, Supporting Information). The serum iron level decreased by 70.86% in ID mice compared with IN mice (Figure S1d, Supporting Information, *p* < 0.001). In addition, the non‐heme iron content in the spleen, liver, and lungs decreased by 96.21% (Figure S1e, Supporting Information, *p* < 0.001), 83.16% (Figure S1f, Supporting Information, *p* < 0.001), and 40.42% (Figure S1g, Supporting Information, *p* < 0.05), respectively. These results indicate the successful establishment of the ID mouse model, as described previously.^[^ ^26^ ^]^

**Figure 1:**
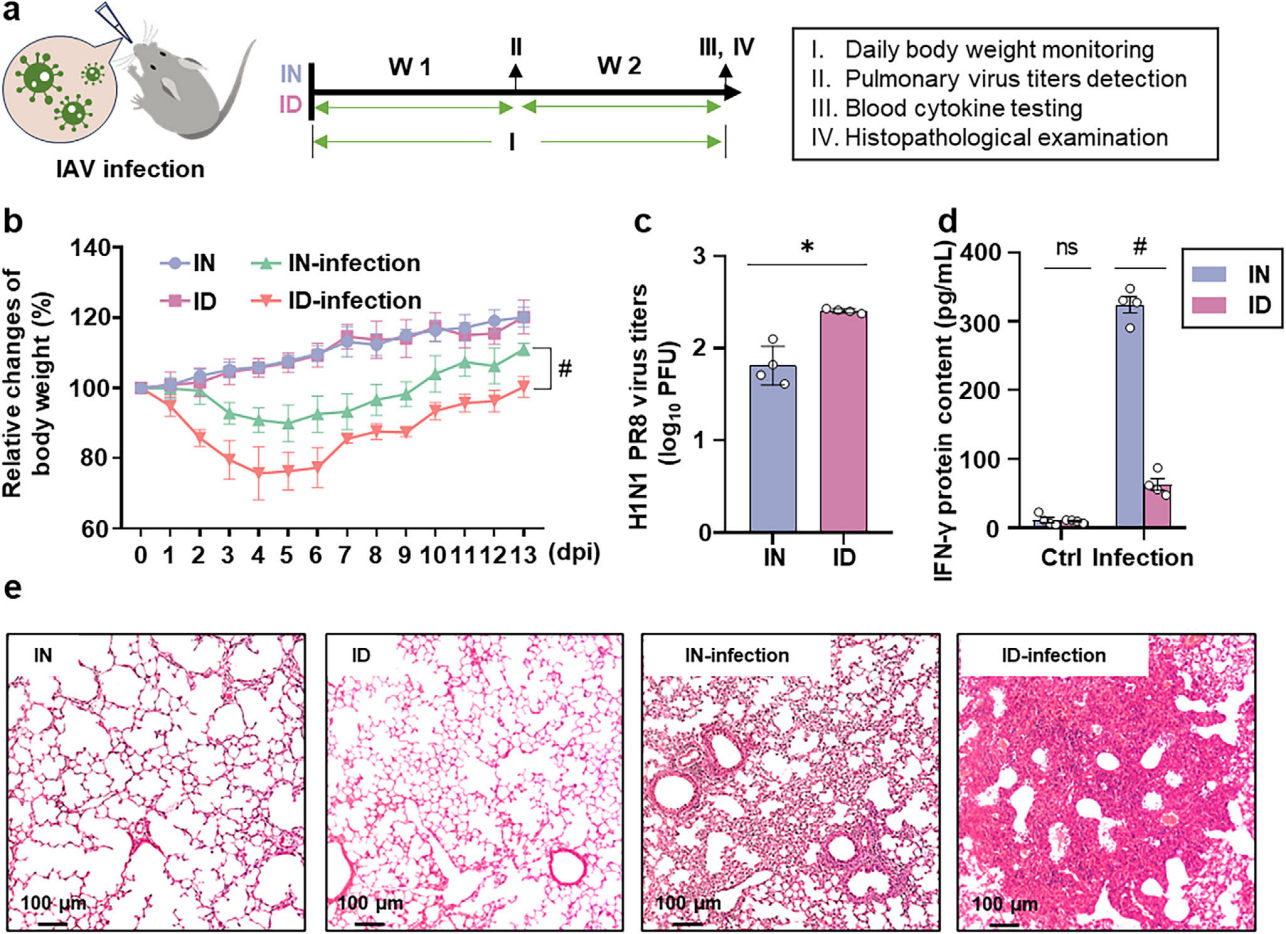
ID exacerbates lung viral infection. a) Timeline and experimental design to evaluate the antiviral ability of mice infected with H1N1 PR8 viruses at 500 plaque‐forming units (PFU) using the nasal instillation method. b) Relative changes in body weight of the mice up to 14 dpi. The body weights were measured daily (*n* = 6). Each mouse was weighed three times, and the average value was used. ID mice show significant reduction in body weight after H1N1 PR8 infection. (c) qRT‐PCR analysis of viral titers in the lungs of mice at 7 dpi (*n* = 3). Viral titers in the lungs of ID mice were increased significantly at 7 dpi. (d) Enzyme‐linked immunosorbent assay (ELISA) was used to measure the protein content of IFN‐γ in the serum of the IN and ID mice at 7 dpi, respectively (*n* = 4). The infection group was infected with the influenza virus by nasal instillation, and the control group was administered the same volume of normal saline. The level of IFN‐γ protein in serum of ID mice was lower than that of normal mice at 7 dpi. (e) Representative hematoxylin and eosin (H&E)‐stained lung sections from IN and ID mice treated with or without the virus for 14 d. ID mice exhibited more severe lung tissue damage at 14 dpi. IN mice received normal iron diet only. *p* < 0.001 (#) relative to the IN mice. IN: iron‐normal mice; ID: iron‐deficient mice.

Thereafter, we infected IN and ID mice with IAV using 500 plaque‐forming units (PFU) of H1N1 PR8 through the intranasal route as previously described and measured the changes in body weight,^[^ ^23^ ^]^ virus titers, and serum cytokines (**Figure** **1**a). By 14 days postinfection (dpi), the body weights of IN and ID mice were reduced by 7.61% and 16.55%, respectively, compared to those of their respective uninfected controls (Figure 1b and *p* < 0.001).

To determine the viral titers in lung tissues, we measured viral matrix protein 1 of the PR8 (PR8‐M1) gene using quantitative real‐time reverse transcription polymerase chain reaction (qRT‐PCR) to determine viral replication and transcription within the cells.^[^ ^23^, ^27^, ^28^ ^]^ The virus titers for each sample was obtained by converting the cycle threshold (Ct) values (namely, the cycle number at which the PCR product exceeds the set threshold) using the standard curve generated from known virus titers (Figure S1h, Supporting Information). The viral titer in the lungs of ID mice was 1.33‐fold higher than that in IN mice at 7 dpi (Figure 1c and p < 0.001). Results of enzyme‐linked immunosorbent assay (ELISA) showed that the content of interferon‐γ (IFN‐γ) protein in serum of infected ID mice was decreased by 80.52%, compared to infected IN mice (Figure 1d and *p* < 0.001). As IFN‐γ, an indicator of antiviral response, acts on multiple mechanisms to inhibit viral replication and spread, its reduced production and the accumulation of virus suggested a decrease in viral clearance as described previously.^[^ ^20^ ^]^ These data were also supported by hematoxylin and eosin (H&E)‐stained lung sections showing more obvious tissue injury in infected ID mice (Figure 1e). Overall, our findings suggest that ID leads to marked impairment of antiviral immune responses, viral clearance, and aggravated lung injury upon pulmonary viral infection.

### 2.2 ID Suppresses DC‐Mediated Anti‐Viral Inflammatory Responses

**Figure 2:**
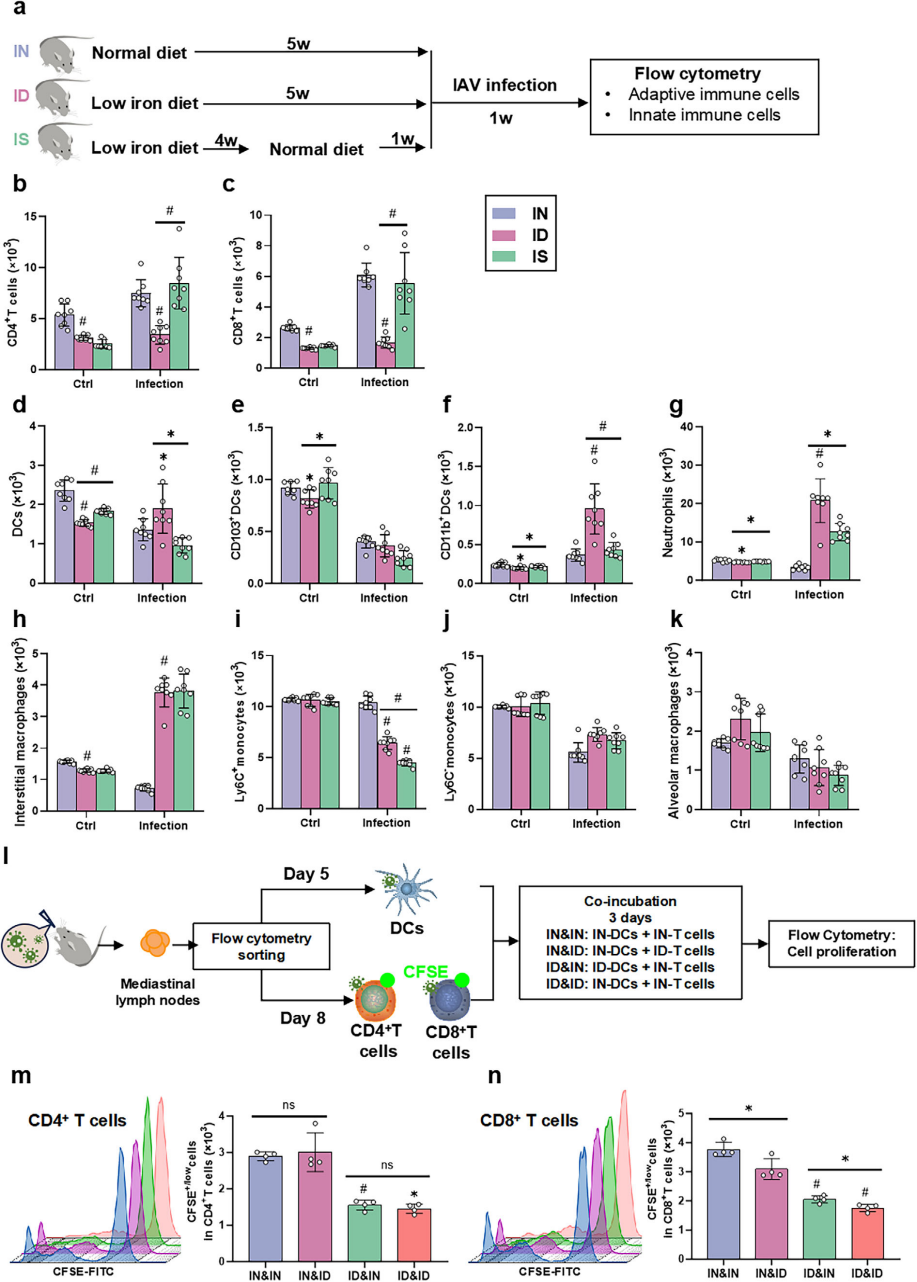
ID increases lung susceptibility to viral infection by impairing DC‐centered adaptive immunity. a) Schematic representation of immune cell analysis in the lungs of IAV‐infected mice at 7 dpi using flow cytometry. IN and ID mice were fed a standard diet containing a normal amount of iron or a low‐iron diet for 5 weeks. Iron‐supplemented (IS) mice were fed a low‐iron diet for the first 4 weeks, then a normal diet for the 5th week, and infected with IAV in the 6th week. b–k) Flow cytometry analyzed of the levels of (b) CD4^+^ T cells (CD3^+^CD19^−^CD4^+^CD8^−^), (c) CD8^+^ T cells (CD3^+^CD19^−^CD4^−^CD8^+^), (d) total DCs (CD45^+^ CD24^+^CD11c^+^MHC II^+^, (e) CD103^+^ DCs (CD45^+^CD24^+^CD11c^+^MHC II^+^ CD103^+^CD11b^−^), (f) CD11b^+^ DCs (CD45^+^ CD24^+^CD11c^+^MHC II^+^CD103^−^CD11b^+^), (g) neutrophils (CD45^+^ Ly6G^+^CD11b^+^), (h) interstitial macrophages (CD45^+^CD24^−^CD11b^+^MHC II^+^CD64^+^), (i) Ly6C^+^ monocytes (CD45^+^CD24^−^CD11b^+^MHC II^−^Ly6C^+^), (j) Ly6C^−^ monocytes (CD45^+^CD24^−^CD11b^+^MHCII^−^Ly6C^−^), and (k) alveolar macrophages (CD45^+^CD170^+^CD11c^+^CD64^+^) collected from lung single‐cell suspensions of IN, ID, and IS mice treated with or without virus after 7 d, respectively (*n* = 8). The infection group was infected with influenza virus by nasal drip, and the Ctrl group was given the same volume of normal saline. *p* < 0.05 (*) and *p* < 0.001 (#) relative to the IN mice. *p* < 0.05 (*) and *p* < 0.001 (#) relative to the ID mice. l) A schematic representation of a model of adaptive immune cell activation mediated by virus‐specific DCs. Lung DCs were sorted by flow cytometry from normal and ID mice infected with influenza virus for 5 d. The proliferation of T lymphocytes was analyzed using flow cytometry after co‐incubation with virus‐specific DCs. m,n) The number of CSFE^+/low^ cells in (m) CD4^+^ T and (n) CD8^+^ T cells after 3 d of stimulation with virus‐specific DCs was determined by flow cytometry (*n* = 4). Virus‐specific DCs derived from ID mice induce lower T‐lymphocyte proliferation. *p* < 0.05 (*) and *p* < 0.001 (#) relative to IN mice. *p* < 0.05 (*) relative to the ID mice. CFSE: carboxyfluorescein diacetate succinimidyl ester. IN: iron‐normal mice; ID: iron‐deficient mice; IS: iron‐supplemented mice; w: week.

To clarify whether ID affects immune cell responses in the lungs after influenza virus infection, we used flow cytometry to measure changes in immune cells from 1 to 8 dpi (Figure S2a–c, Supporting Information). The results showed that in the early stages of infection (from 1 to 3 dpi), the number of innate immune cells in the lungs, such as neutrophils, monocytes, alveolar macrophages, interstitial macrophages, and DCs, increased (Figure S2c, Supporting Information). Considering that the expansion of virus‐specific CD4^+^ and CD8^+^ T cells is key to immune responses against IAV infection we analyzed the changes in immune cell subpopulations in response to virus infection in ID mice by flow cytometry (**Figure** **2**a).^[^ ^29^, ^30^ ^]^ We found that in the late stage of influenza virus infection (from 4 to 8 dpi), the innate immune cells in the lungs returned to normal levels, while CD4^+^ and CD8^+^ T cells began to proliferate, reaching a peak at ≈7 dpi (Figure S2c, Supporting Information). To clarify whether the change in iron supplementation affected the outcomes of influenza virus infection, we fed ID mice a normal diet for 7 d to replenish their iron content, which was named the iron‐supplemented (IS) group (Figure 2a). We found that the iron content in the liver, spleen, and lung tissues of IS mice was restored, which was 3.03 times (Figure S3a, Supporting Information, *p* < 0.001), 6.79 times (Figure S3b, Supporting Information, *p* < 0.001), and 1.14 times (Figure S3c, Supporting Information, *p* < 0.05) those of the mice in the ID group, respectively. In addition, hemoglobin content returned to the normal range after iron supplementation (Figure S3d, Supporting Information), indicating that these ID and IS mouse models can be used to analyze the influence of iron supply on antiviral ability. Compared to ID mice, the viral titer in the lungs of IS mice decreased by 6.47% (Figure S3e, Supporting Information, *p* < 0.05). Finally, H&E staining indicated that compared to ID mice, the damaged areas in the lungs of IS mice were reduced (Figure S3f, Supporting Information). These results indicate that iron supplementation can effectively enhance the resistance to influenza virus infection.

To further confirm that the change in iron supplementation is a key factor affecting pulmonary immune cell responses to influenza virus infection, we analyzed the subpopulations of pulmonary immune cells in the absence of virus infection. Compared with IN mice, the numbers of CD4⁺ T cells, CD8⁺ T cells, total DCs, CD103⁺ DCs, CD11b⁺ DCs, neutrophils, and interstitial macrophages in ID mice were decreased by 41.77%, 50.95%, 34.92%, 11.47%, 17.25%, 9.07%, and 18.12% (Figure 2b–h and *p* < 0.05) respectively, due to ID‐limited immune cell development in bone marrow as described.^[^ ^7^, ^8^ ^]^ The iron supplementation enhanced the numbers of total DCs, CD103⁺ DCs, CD11b⁺ DCs, and neutrophils in IS mice by 19.35%, 18.58%, 8.56%, and 4.01% compared with ID mice, respectively (Figure 2d–f and *p* < 0.05) The results indicated that a reduction in pulmonary iron levels affects the levels of various cell types, and that cell numbers can be restored after iron supplementation.

Subsequently, we analyzed the effects of iron supplementation on the number of immune cells after influenza viral infection. At 7 dpi, compared with infected‐IN mice, the numbers of CD4⁺ T cells, CD8⁺ T cells, and Ly6C⁺ monocytes in infected‐ID mice decreased by 54.47% (Figure 2b and *p* < 0.001), 72.54% (Figure 2c and *p* < 0.001), and 37.98% (Figure 2i and *p* < 0.001) respectively. In normal mice at 7 dpi, the numbers of both CD4⁺ T cells and CD8⁺ T cells showed an upward trend (Figure S2c, Supporting Information). In ID mice, the numbers of CD4⁺ T cells and CD8⁺ T cells did not increase. These data demonstrated that ID impedes the activation of CD4⁺ and CD8⁺ T cells. In addition, the decrease in the number of Ly6C⁺ monocytes was presumably due to their differentiation into DC and macrophages.^[^ ^31^ ^]^ Moreover, the numbers of total DCs, CD11b⁺ DCs, neutrophils, and interstitial macrophages increased by 39.76% (Figure 2d and *p* < 0.05), 163.82% (Figure 2f and *p* < 0.001), 520.81% (Figure 2g and *p* < 0.001), and 422.04% (Figure 2h and *p* < 0.001), respectively. After iron supplementation, compared with infected ID mice, the numbers of CD4⁺ and CD8⁺ T cells in infected IS mice increased by 148.74% and 231.78% (Figure 2b,c and *p* < 0.001) respectively. Moreover, the number of total DCs, CD11b⁺ DCs, neutrophils, and Ly6C⁺ monocytes decreased by 49.39% (Figure 2d and *p* < 0.05), 55.02% (Figure 2f and *p* < 0.001), 39.56% (Figure 2g and *p* < 0.05), and 30.51% (Figure 2i and *p* < 0.001), respectively. By analyzing the above results, we found that compared with normal iron levels, ID led to a significant decrease in the numbers of CD4⁺ and CD8⁺ T cells after influenza virus infection. Normally, the numbers of these cells reach their peak at 7 dpi and begin to clear the virus from the lungs. When the proliferation of CD4⁺ and CD8⁺ T cells is restricted in ID mice, clearance mediated by these cells toward virus would be reduced, resulting in a large‐scale and prolonged infiltration of innate immune cells that exacerbates damage to the lung tissue.^[^ ^20^ ^]^ After iron supplementation, the numbers of CD4⁺ T cells, CD8⁺ T cells, and DCs in IS mice returned to normal levels, and the viral infection receded, indicating that the reduction in the numbers of CD4⁺ and CD8⁺ T cells might be a contributing factor to the exacerbation of lung injury after influenza virus infection during ID.

To further clarify the influence of ID on DC‐mediated T cell activation, we isolated DCs, CD4^+^ T cells, and CD8^+^ T cells from the mediastinal lymph nodes of infected mice at 5 and 8 dpi (Figure S4a, Supporting Information), because lung DCs migrate to the nearest mediastinal lymph nodes and reach peak levels at ≈3–5 dpi, and the peak of T cell activation and proliferation occurs ≈6–8 dpi.^[^ ^32^, ^33^ ^]^ The purities of IN‐DC, ID‐DC, IN‐CD4^+^ T, ID‐CD4^+^ T, IN‐CD8^+^ T, and ID‐CD8^+^ T cells were 86.80%, 84.10%, 83.50%, 84.40%, 80.80%, and 82.10%, respectively (Figure S4b, Supporting Information).

Furthermore, the proliferation of carboxyfluorescein diacetate succinimidyl ester (CFSE)‐labeled CD4^+^ and CD8^+^ T cells was analyzed by flow cytometry after co‐incubation with isolated DCs for 3 d (Figure 2l). The proliferative ability of CD4⁺ T cells was comparable between the IN‐and ID‐DCs (Figure 2m). Importantly, we found that IN‐DCs stimulated the proportion of CFSE^⁺/low^ cells, namely the proliferating cells, in IN‐CD4⁺, ID‐CD4⁺, IN‐CD8⁺, and ID CD8⁺ T cells increased by 45.96%, 45.42%, 40.20%, and 37.64%, respectively, relative to cells upon ID‐DC co‐incubation (Figure 2m,n and *p* < 0.001). Compared with IN‐DC‐stimulated IN‐CD8⁺ T cells, the proportion of CFSE^⁺/low^ in ID‐CD8⁺ T cells upon co‐incubation with IN‐DCs or ID‐DCs were decreased by 17.76% or 14.51%, respectively (Figure 2n and *p* < 0.05). These data suggested that changes in iron levels significantly affected the ability of DCs to present antigens and activate T cells.

### 2.3 ID Decreases the Activation of Virus‐Specific T Cells via Reducing Antigen‐Presentation of DCs

**Figure 3:**
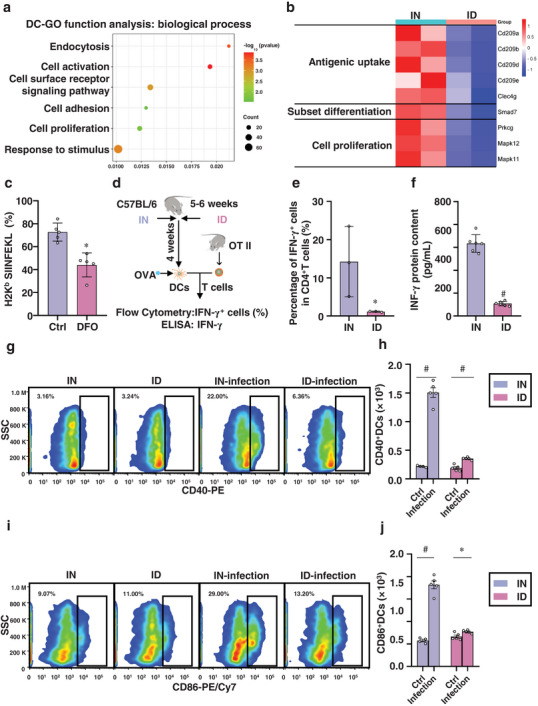
DCs derived from ID mice have a reduced ability to activate T cells. a) GO function analysis of DCs. ID inhibits DC endocytosis, cell activation, cell surface receptor signaling pathway, cell adhesion, cell proliferation, and response to stimulation. b) Differential gene analysis of DCs. ID downregulated genes involved in antigen uptake, subset differentiation, and cell proliferation in DCs. c) The percentage of H2Kb SIINFEKL^+^ cells in DFO (2 µm)‐treated and untreated BMDCs after 24 h (*n* = 3). ID inhibits MHC class I antigen presentation by DCs. d) A schematic diagram illustrating the establishment of the ID animal model, and analysis of the percentage of IFN‐γ^+^ CD4^+^ T cells and content of IFN‐γ protein induced by OVA antigen‐pulsed ID‐derived DCs. e) The percentage of IFN‐γ^+^CD4^+^ T cells after co‐culturing with sorted DCs (*n* = 3) shows that ID inhibits MHC class II antigen presentation by DCs. f) ELISA was used to measure the protein levels of IFN‐γ in the culture after co‐culturing with sorted DCs for 48 h (*n* = 6). CD4^+^ T cells activated by ID DCs produced lower levels of IFN‐γ. g–j) Flow cytometry analysis of the number of (g,h) CD40^+^ DCs and (i,j) CD86^+^ DCs from lung single‐cell suspension obtained from IN and ID mice treated with or without virus at 7 dpi, respectively (*n* = 5). *p* < 0.05 (*) and *p* < 0.001 (^#^) relative to the IN mice. DFO: desferrioxamine; IN: iron‐normal mice; ID: iron‐deficient mice.

The spleen is the primary organ where lung‐originating DCs activate T cells to clear influenza virus from the lungs.^[^ ^34^ ^]^ Researches have indicated that following influenza virus infection, pulmonary DCs migrate to the mediastinal lymph nodes and subsequently to the spleen to engage virus‐specific T cells, which exhibit an extended lifespan compared to T cells within the lymph nodes.^[^ ^34^ ^]^ Therefore, to further illustrate the influence of ID on DC functions, we isolated DCs from a single‐cell suspension of spleen collected from IN and ID mice using immunomagnetic beads and subjected them to comparative RNA‐seq analyses. RNA‐seq analysis revealed that 258 genes were downregulated (|log2 fold change| ≥ 1 and *p <* *0.05*) in DCs derived from ID mice. Gene Ontology (GO) function analysis revealed that these downregulated genes were mainly enriched in biological processes, such as endocytosis, cell activation, cell surface receptor signaling pathway, cell adhesion, and cell proliferation (**Figure** **3**a). Among them, CD209 family members encode C‐type lectin receptors on antigen‐presenting cells and are involved in functions such as adhesion, migration, signaling, and antigen uptake/presentation.^[^ ^35^ ^]^ Clec4 g is involved in antigen uptake and internalization.^[^ ^36^ ^]^ Therefore, the significant downregulation of CD209a, CD209b, CD209d, CD209e, and Clec4 g in DCs derived from ID mice indicated a decreased ability for antigen uptake and presentation (Figure 3b). Moreover, the downregulation of the Smad7 gene resulted in an increase in the ratio of conventional type 1 DCs and a decrease in the ratio of conventional type 2 DCs (Figure 3b) because its expression controls the differentiation of splenic DC subsets,^[^ ^37^ ^]^ and DC‐specific Smad7 deficiency leads to increased expression of the transcription factors Batf3 and IRF8, resulting in an elevated frequency of conventional type DCs in the spleen.^[^ ^37^ ^]^ ID also led to the downregulation of proliferation‐related genes such as Prkcg, Mapk11, and Mapk12 (Figure 3b), suggesting that the downregulation of proliferation‐related genes caused by ID may contribute to a reduced number of DCs.

To verify these sequencing results (Figure 3b), we first analyzed the antigen presentation ability of bone marrow‐derived DCs (BMDCs) by treatment with desferrioxamine (DFO). The OVA_257‐264_ peptide was used as a model major histocompatibility complex class I (MHC‐I) I‐specific antigen.^[^ ^38^ ^]^ The flow cytometry analyses showed that the percentage of H2Kb SIINFEKL^+^ CD11c^+^ within DCs, namely antigen‐laden DCs, was significantly reduced by 39.45% in BMDCs derived from ID mice compared with that in the control group (Figure 3c; Figure S5a, Supporting Information, *p <* 0.05). Moreover, DCs isolated from the spleens of IN or ID mice were pre‐loaded with OVA_323‐339_ MHC‐II‐targeting peptide and then were incubated with splenic cells from OT‐II mice to assess their ability to present antigens. After 12‐h culture, the percentage of IFN‐γ^+^ cells was significantly reduced by 87.13% in CD4^+^ T cells pulsed by ID mice‐derived DCs compared with that from IN mice (Figure 3d,e; Figure S5b, Supporting Information, *p <* 0.05). In addition, after 3‐day co‐incubation of DCs‐derived from ID mice and spleen cells, the level of IFN‐γ protein in the medium was significantly reduced by 82.88% compared with that in a DC‐spleen cell mixture from IN mice (Figure 3f and *p* *<* 0.001). CD40 and CD86 play crucial roles in T‐cell activation by DCs.^[^ ^39^ ^]^ The binding of CD40 on the surface of DCs to CD40L on T cells activates the DC signaling pathway, enhances the antigen‐presenting ability of DCs, promotes the secretion of interleukin (IL)‐12, guides the differentiation of naïve CD4⁺ T cells into Th1 cells, and strengthens cell‐mediated immunity. As a co‐stimulatory molecule, CD86 binds to CD28 on T cells to provide a second signal that drives T cell activation, proliferation, and cytokine secretion.^[^ ^39^ ^]^ The expression levels of the surface costimulatory molecules CD40 and CD86 on lung DCs, characterized by the number of CD40^+^ DCs and CD86^+^ DCs, increased 5.91‐fold (Figure 3g,h and *p* < 0.001) and 2.15‐fold (Figure 3i,j and *p* < 0.001), respectively, in IN mice after infection. In contrast, these increases were reduced by 0.88‐fold (Figure 3g,h *and* *p* < 0.001) and 0.16‐fold (Figure 3i,j and *p* < 0.05), respectively, in DCs derived from ID mice after infection, indicating a hampered ability of DCs in antigen presentation in ID mice. Together, these results indicate that ID led to decreased antigen presentation ability of DCs, leading to decreased ability to induce adaptive immune cell differentiation and consequently weakened anti‐viral ability.

### 2.4 ID Hampers DC Development and Function at the MDP Stage

**Figure 4:**
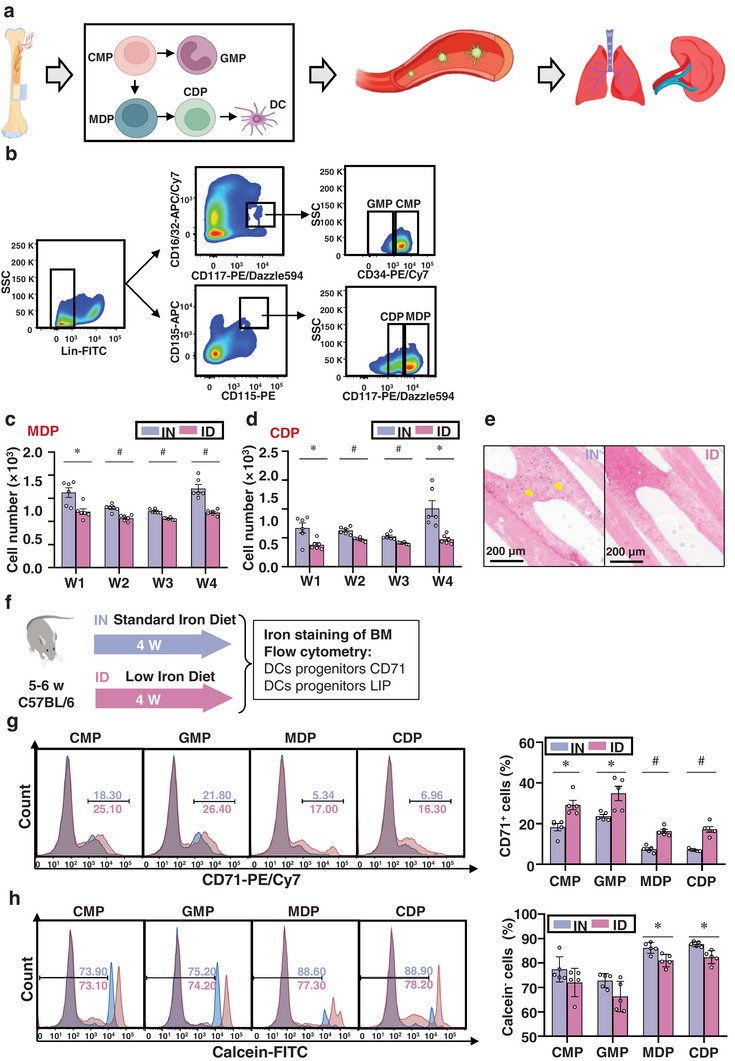
The number of MDPs and CDPs in bone marrow of low‐iron mice is reduced. a) Schematic representation of differentiation process of DC progenitors. DCs develop in the bone marrow, undergo the CMP, MDP, and CDP stages to differentiate into DCs, and reside in tissues such as the lung and spleen through the blood circulation. b) Graded analysis of DC progenitors in gated cells (5 × 10^5^) collected from IN and ID mice through flow cytometry. c,d) Flow cytometry analysis of the number of MDPs (Lin^−^CD115^+^CD135^+^CD117^Hi^, c) and CDP (Lin^−^CD115^+^CD135^+^CD117^Lo^, d) derived from IN and ID groups, respectively (*n* = 5). The numbers of MDPs and CDPs were significantly reduced in ID mice. e) Tissue iron staining of the bone marrow tissue sections from IN and ID mice. Blue spots (yellow arrow) show deposited iron in the bone marrow sections. ID mice have almost no detectable presence of iron in the bone marrow. f) The levels of CD71 and LIP in DC progenitors were measured by flow cytometry. g) Flow cytometry analysis of the percentage of CD71^+^ cells in DC progenitor cells derived from IN and ID mice, respectively (*n* = 5). CD71 levels on the surface of DC progenitors were all significantly increased. h) Flow cytometry analysis of the percentage of calcein‐negative cells in DC progenitor cells derived from IN and ID mice, respectively (*n* = 5). Intracellular LIP levels were significantly reduced in the DC progenitors, MDPs, and CDPs. The IN mice received a normal iron diet only. *p* < 0.05 (*) and *p* < 0.001 (#) relative to the IN mice. CDP: common DC precursors; Lin: lineage cocktail including CD3, CD45R, CD11b, TER‐119 and Gr‐1. IN: iron‐normal mice; ID: iron‐deficient mice; LIP: labile iron pool; MDP: monocyte DC progenitor.

To determine whether ID specifically affected the number and function of DCs, we screened DCs in a single‐cell suspension of the lung, spleen, bone marrow, and blood, because DCs are derived from the bone marrow and mature in the lung and spleen (**Figure** **4**a). Interestingly, a significant decrease ranging from 14.98%–63.57% was detected in these organs of ID mice (Figure S6a–d, Supporting Information, *p <* 0.001), compared to IN mice, especially in bone marrow where a 35.26% decrease was observed. In the bone marrow, hematopoietic stem cell‐derived multipotent progenitors undergo stages of differentiation to produce lineage‐restricted progenitors of lymphocytes, myeloid cell‐common lymphoid progenitors, and common myeloid progenitors (CMPs).^[^ ^40^ ^]^ CMPs gave rise to granulocyte–monocyte progenitors (GMPs) and MDPs. Initial studies on the development of DCs from bone marrow progenitors identified a population referred to as common DC precursors (CDPs). Developing of MDPs and CDPs gives rise to DCs.^[^ ^40^ ^]^ Therefore, we analyzed the changes in the populations of CMP, GMP, MDP, and CDP in the bone marrow after 4 weeks in ID mice (Figure 4b). In contrast to CMP and GMP (Figure S6e,f, Supporting Information), the number of MDPs and CDPs was significantly reduced at the beginning of the 1st week of the low‐iron diet. After 1, 2, 3, and 4 weeks of the low‐iron diet, the number of MDP decreased by 25.57%, 16.96%, 13.22%, and 30.35% (Figure 4c and *p* *<* 0.001), respectively. The number of CDP decreased by 43.29%, 24.65%, 22.39%, and 53.09% (Figure 4d and *p* *<* 0.05), respectively, compared with IN mice, indicating a disorder in DC development caused by ID.

Images of iron‐stained sections of bone marrow tissue showed decreased iron storage in ID mice compared to IN mice (Figure 4e). To clarify whether the decreased number of DC progenitor cells was caused by deficient intracellular iron required for their development, we further isolated DC progenitor cells by flow cytometry. Iron absorption requires the cooperation of many proteins, including the transferrin receptor (CD71), which is responsible for the transport of extracellular iron to the intracellular space, followed by endocytosis, acidification, release, and displacement. When iron is deficient, cells enhance the expression CD71 via the iron regulatory protein (IRP)‐iron‐responsive element (IRE) regulatory system.^[^ ^41^ ^]^ Additionally, iron enters the cytoplasm and forms a labile iron pool (LIP), which can be directly used by cells.^[^ ^41^ ^]^ Therefore, we measured CD71 and LIP levels in DC progenitor cells from IN and ID mice (Figure 4f). We found that the proportion of CD71^+^ cells within CMPs, GMPs, MDPs, and CDPs from ID mice increased by 59.65% (*p <* 0.05), 47.63% (*p <* 0.05), 118.99% (*p <* 0.001), and 143.59% (*p <* 0.001), respectively, compared to IN mice (Figure 4g), suggesting a compensatory requirement for iron intake in response to reduced intracellular iron content, which is consistent with previous reports.^[^ ^42^ ^]^ Next, we measured the content of LIP in DC progenitors by analyzing the percentage of calcein‐negative cells labeled with calcein‐FITC. The content of LIP in MDPs and CDPs of ID mice was significantly decreased by 9.45% (*p <* 0.05) and 12.63% (*p <* 0.05), respectively, relative to that of DCs derived from IN mice (Figure 4h), indicating a disorder in the storage of available iron in the lineage of DCs from the bone marrow. Taken together, these results indicate that ID leads to an iron‐dependent decrease in DC progenitor cells, especially MDPs and CDPs.

### 2.5 ID Causes MDP Inhibition by Increasing the Disorder Risk of Cell Migration, Cell Proliferation, and Inducing Cell Apoptosis

**Figure 5:**
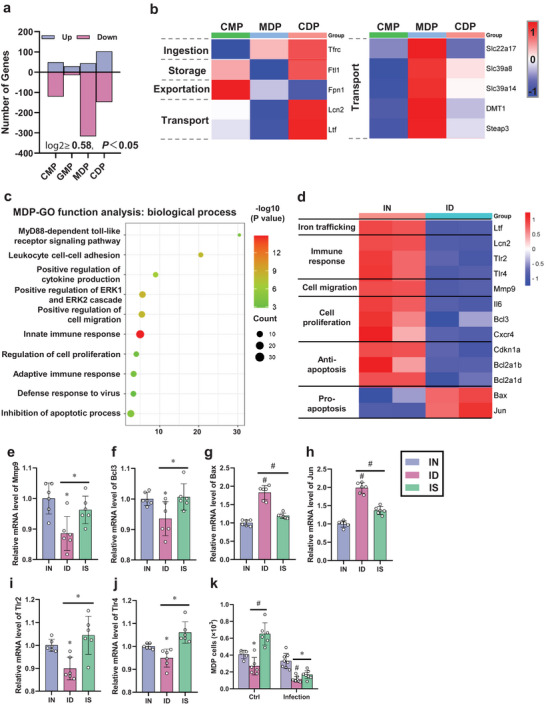
ID decreased immune response, cell migration, and proliferation, and increased the potential for apoptosis in MDPs. a) RNA‐seq analysis results showing upregulation (blue) and downregulation (pink) of genes in IN and ID mice‐derived DC progenitor cells in the bone marrow (*n* = 2). ID resulted in more downregulated genes in MDPs. b) Expression levels of iron‐related genes in DC progenitor cells from IN mice (*n* = 2). Iron‐related genetic changes in MDPs result in higher iron requirements. c) Gene Ontology (GO) enrichment analysis of annotated downregulated genes in MDPs from IN and ID mice. d) Heatmap showing differentially expressed genes in MDPs categorized by biological processes. The columns represent samples (*n* = 2 per treatment group). e–j) Changes in the expression of key genes in the sequencing results verified by qRT‐PCR (*n* = 6). k) Flow cytometry analysis of changes in MDP cells from bone marrow cell suspensions obtained from IN, ID, and IS mice treated with or without the virus measured 7 d postinfection (*n* = 6 or 8). *p* < 0.05 (*) and *p* < 0.001 (#) for comparisons as indicated by the horizontal bars, or relative to IN mice. IN: iron‐normal mice; ID: iron‐deficient mice; IS: iron‐supplemented mice.

To elucidate the mechanism by which ID causes significant inhibition of MDPs and the subsequent inhibition of DC development. CMPs, GMPs, MDPs, and CDPs were derived from the bone marrow of IN and ID mice, sorted by flow cytometry, and subjected to RNA‐seq analysis. The purities of CMPs, GMPs, MDPs, and CDPs were 90.90%, 81.30%, 97.80%, and 85.10%, respectively, which satisfied the standards of the specific sequencing analysis (Figure S7, Supporting Information). First, we performed a statistical analysis of differentially expressed genes (DEGs) in DC progenitor cells from ID mice. RNA‐seq results showed that there were 170, 44, 361, and 251 DEGs in CMPs, GMPs, MDPs, and CDPs, respectively (**Figure** **5**a, |log2 fold change| ≥ 0.58 and *p <* 0.05). ID led to the upregulation of 121 genes and downregulation of 49 genes in CMPs, the upregulation of 15 genes and downregulation of 29 genes in GMPs, the downregulation of 317 genes and upregulation of 44 genes in MDPs, and the downregulation of 148 genes and upregulation of 103 genes in CDPs. Compared with CMPs, GMPs, and CDPs, ID induced more gene changes in MDPs, which may have contributed to the reduction in MDP levels. Therefore, we focused on the expression of iron‐related genes in each DC progenitor cell line (Figure 5b). Consistent with Figure 4g, the genes in MDPs and CDPs related to iron uptake (Tfrc) and iron transport were significantly upregulated, whereas the iron efflux gene Fpn1 (ferroportin 1) downregulated compared to that in CMPs. Compared to CDPs, genes related to iron storage were significantly downregulated in MDPs, suggesting that MDPs have a higher demand for available iron compared to CDPs. Overall, these findings suggest that the MDP stage is iron‐sensitive and susceptible to ID during DC development.

Because most DEGs in MDPs were downregulated, we further analyzed how these DEGs contributed to the reduction in the MDP population. GO enrichment analysis revealed that these downregulated genes were primarily enriched in processes related to cell expansion (e.g., upregulation of cell proliferation and inhibition of apoptosis), immune cell maturation, and relative potential in inflammatory responses (e.g., MyD88‐dependent toll‐like receptor signaling pathway, leukocyte cell–cell adhesion, positive regulation of cytokine production, positive regulation of ERK1 and ERK2 cascade, positive regulation of cell migration, innate immune response, adaptive immune response, and defense response to virus) (Figure 5c).

In the steady state, hematopoietic stem/progenitor cells maintain hematopoietic homeostasis through proliferation, self‐renewal, migration, and responses to emergencies and damage.^[^ ^43^ ^]^ Cell adhesion and apoptotic signaling play critical roles in these processes.^[^ ^44^, ^45^ ^]^ Therefore, we proposed that dysregulation of these genes in the MDPs of ID mice may lead to impaired proliferation and migration, resulting in reduced MDP numbers.^[^ ^46^ ^]^ Consequently, ID may lead to a decreased number and impaired antigen presentation by DCs and a relative disorder in antiviral T cell activation.

Therefore, we conducted a comparative analysis of DEGs and iron‐related genes to elucidate the mechanisms underlying MDP inhibition (Figure 5d). Significant downregulation of *Lcn2*, *MMP‐9*, *IL‐6*, and *BCL3* gene expression was found in MDPs from ID mice (Figure 5d–f). Lcn2 can form a complex with MMP‐9, which enhances its enzymatic activity and promotes cell migration.^[^ ^47^, ^48^, ^49^ ^]^ Decreased IL‐6 levels hinder hematopoietic stem cell expansion, whereas IL‐6‐induced BCL3 expression is associated with cell proliferation.^[^ ^50^, ^51^, ^52^, ^53^ ^]^ Additionally, ID resulted in DNA damage and upregulation of genes such as Bax and Jun (Figure 5d,g,h), contributing to increased apoptosis.^[^ ^54^, ^55^ ^]^ Impaired cell migration, proliferation, and increased apoptosis might collectively contribute to the decrease in MDP numbers, as described.^[^ ^43^, ^56^ ^]^ In addition, we showed that both CDPs and offspring DCs express pathogen‐recognition receptors such as Toll‐like receptor 2 (TLR2) and TLR4,^[^ ^57^ ^]^ which are inherited from MDPs, and that the expression of TLR2 and TLR4 in MDPs was significantly reduced in ID mice (Figure 5d,i,j). ID inhibits TLR4 signaling.^[^ ^58^ ^]^ In addition, TLR2–MyD88 signaling plays a key role in the clonal expansion of CD8^+^ T cells after viral infection. Effector CD8^+^ T cells lacking TLR2–MyD88 signaling fail to expand, survive, and differentiate into memory cells.^[^ ^59^ ^]^ Finally, we detected changes in the number of MDPs after iron supplementation (Figure 5k). Compared with ID mice, the number of MDPs in IS mice increased significantly by 141.76% (*p* < 0.001) and 47.01% (*p* < 0.001) before and after infection, respectively. The expression levels of *Mmp9, Bcl3, Bax, Jun, Tlr2*, and *Tlr4* also returned to normal (Figure 5e–j). Consequently, we concluded that ID diminished the number and immune‐related potential of DCs by impeding the migration, proliferation, and immune‐related gene expression of MDP, which potentially resulted in the ineffectiveness of pulmonary DCs in activating the antiviral immune response.

## 3 Conclusion

Inspired by epidemiological and clinical data indicating increased injury severity and poor prognosis in patients with ID upon viral infection, we investigated the underlying biological mechanisms. Mounting evidence suggests that when the body is in an iron‐deficient state, the intracellular heme concentration decreases. This reduction activates heme‐regulated eIF2α kinase (HRI), along with its downstream phosphorylated eukaryotic initiation factor 2α (eIF2αP) and activating transcription factor 4 (ATF4). In mouse models lacking eIF2αP or ATF4, erythrocyte differentiation is hindered, and intracellular oxidative stress levels increase significantly. These changes ultimately lead to a decrease in the number of red blood cells and ineffective erythropoiesis.^[^ ^60^ ^]^ Meanwhile, the ID state prompts an increase in erythropoietin (Epo) levels. The increase in Epo further activates the phosphatidylinositol 3‐kinase/protein kinase B (PI3K/AKT) and the target of rapamycin complex 1 (mTORC1) signaling pathways. In mice with defective HRI–eIF2αP–ATF4 signaling, mTORC1 signaling is abnormally elevated. This abnormal elevation inhibits erythrocyte differentiation, resulting in an increase in the number of ineffective red blood cells and a decrease in the total number of red blood cells.^[^ ^60^ ^]^ These changes lead to a decline in hemoglobin levels, reducing the ability to transport oxygen to cells throughout the body, which can cause cell hypoxia. Simultaneously, a hypoxic state may affect the differentiation and maturation of immune cells, further weakening the immune system's defense against viral infections.^[^ ^61^ ^]^ In this study, we found a significant decrease in CD4^+^ T cell, CD8^+^ T cell, and DC cell counts in response to a diet low in iron, even without viral infection. Our results suggest that systemic ID affects the proliferation and migration of MDPs in the bone marrow, resulting in a decrease in the number of peripheral DCs (Figure 5a,d). These findings may also explain the iron‐restricted proliferation of T cell progenitor cells in the bone marrow, similar to the adverse effects caused by ID on neutrophils,^[^ ^7^, ^8^ ^]^ although more work on the influence of ID on the T cell developmental trajectory is required in the future. Moreover, we found that the number of activated T cells that rely on DC‐stimulated proliferation was significantly decreased in ID mice (Figure 2m,n), indicating that ID also hampers T cell‐mediated defense against viruses and leads to increased susceptibility to other pathogens. Given the high incidence and global impact of influenza,^[^ ^62^ ^]^ we focused on exploring the pathogenesis of ID in relation to influenza virus infection. We observed impaired function in an ID mouse model infected with influenza virus, which leads to a reduced resistance to infection, resulting in severe lung damage. Mechanistic investigations revealed that ID caused downregulation of genes associated with DC antigen uptake, resulting in decreased antigen uptake and presentation capability. Upon further investigation of DC development, a notable decrease in the MDP population was observed. MDP are particularly vulnerable to ID, which under reduced migration, proliferation, and immune‐related gene expression. This may ultimately lead to a decline in MDP number and impaired functionality, indicating that the effect of ID on DCs begins in the bone marrow. Furthermore, we found that the hemoglobin level in iron‐supplemented ID mice returned to normal levels, and the viral infection receded (Figure S3d, Supporting Information). These findings underscore the significance of ID in infection defense and the improvement of treatment strategies. Interventions targeting ID are expected to effectively enhance DC functionality, boost immune defenses, mitigate infection risks, and contribute to disease prevention and treatment, thereby offering more efficacious strategies for promoting overall health.

## 4 Experimental Section

### Animal Experimentation

Female mice were used because the disease was more prevalent in females. Four‐week‐old pathogen‐free C57BL/6N mice were purchased from Beijing Vital River Laboratory Animal Technology Co., Ltd. (Beijing, China). OT‐II transgenic mice maintained on a C57BL/6N background were provided by Dr. Hai Qi (Tsinghua University, China). All animals were placed in an environment of 20 °C and ≈55% of relative humidity. All animals were provided with sufficient food and water. To establish the ID mouse model, 4‐week‐old female mice were raised on a low‐iron diet (Beijing Yicheng Technology Co., Ltd. (China), 5.93 ppm, Figure S1b, Supporting Information) for 4 weeks.

### Study Approval

All animal experimental designs and protocols were approved by the Animal Ethics Committee of the Research Center for Eco‐Environmental Sciences, Chinese Academy of Sciences. The ethical approval number was AEWC‐RCEES‐2020001. All infection protocols were performed in strict accordance with the standards of the Institute of Microbiology at the Chinese Academy of Sciences.

### Blood Routine Examination

Peripheral blood was collected and 20 µL was diluted (1:100) with a standard dilution buffer, followed by complete blood count analysis on a hematology analyzer (Nihon Kohden, Japan).

### Tissue Iron Assessment and Histological Examination

Tissue non‐heme iron mass was assessed as previously described.^[^ ^63^ ^]^ Histological examination of the tissue specimens was performed with hematoxylin and eosin (H&E) according to the standard protocol, and iron staining was carried out using Prussian blue staining, as described previously.^[^ ^63^ ^]^

### Viral and Mouse Infection Model

To construct a mouse model of influenza virus infection, the mouse‐adapted human H1N1 PR8 virus was propagated and purified as previously described.^[^ ^23^, ^64^ ^]^ Viral titers were assayed through the plaque assay. The H1N1 PR8 virus was quantified using qRT‐PCR. The mice were intranasally administered the virus at 500 PFU after being anesthetized with pentobarbital sodium. At appropriate time‐points post‐infection, lung tissues were harvested. Briefly, viral genomic RNA was extracted from lung tissues using a Viral RNA Rapid Extraction Kit (Zomanbio, China) according to the manufacturer's instructions. The extracted RNAs was reverse‐transcribed into cDNAs using Script RT All‐in‐One Mix (StarLighter, China). Gene expression levels were determined using an Mx3005P qRT‐PCR instrument (Bio‐Rad, USA) with the primer sequences listed in Table S1 (Supporting Information). For qRT‐PCR detection of virus titers, a series of ten‐fold serial dilutions of the virus stock were prepared, ranging from 10.0 to 1.0 × 10^8^ PFU. The cycle threshold (Ct) values of these dilutions were measured by qRT‐PCR. A standard curve was generated by plotting the log of known viral titers against their corresponding Ct values. The Ct values of the cDNA samples from infected lung tissues were measured using the same qRT‐PCR conditions. By comparing these sample Ct values with the respective standard curves, the influenza virus load in the lungs after infection was accurately determined, providing a quantitative measure of the viral presence in the lung tissue.

### Cytokine Content Determination

After 7 d of infection, blood samples were obtained by enucleation of the eyeball, and serum was obtained by centrifugation at 3000 × g for 15 min. The concentrations of IFN‐γ in the serum and cell culture medium were measured using the QuantiCyto Mouse IFN‐γ ELISA kit (Neobioscience, China), according to the manufacturer's instructions.

### Immune Cell Population Detection

After 7 d of viral infection, mouse lung and spleen tissue were collected and subjected to single‐cell suspension collection. After being washed and diluted with phosphate‐buffered saline (PBS, pH 7.4, Solarbio, China) containing 0.1% fetal bovine serum (FBS; Gibco, USA), the cells (2 × 10^6^) were blocked with TruStain fcX (anti‐mouse CD16/32, Biolegend Inc.) at 4 °C for 15 min and subjected to surface staining with an array of fluorescent‐labeled antibodies (Abs) at 4 °C for 30 min protected from light. All Abs were used as 0.1 µg per sample in 50 µL staining solution. The stained cells were analyzed by flow cytometry (Attune NxT, Thermo Fisher Scientific). Detailed information on the Abs used in flow cytometry is provided in Table S2 (Supporting Information).

### SIINFEKL Assay

BMDCs were prepared and cultured as follows. In brief, bone marrow cells were flushed from the tibia and femurs with PBS (Solarbio) through a 70 µm cell strainer. The cells were suspended in RPMI 1640 medium (Gibco) supplemented with 10% FBS (Gibco), 5% penicillin/streptomycin (Gibco), 20 ng mL^−1^ GM‐CSF (PeproTech, USA) and 10 ng mL^−1^ IL‐4 (PeproTech), then the cells were incubated at 37 °C and 5% CO_2_ for 7 d and medium was replaced every 2 d. BMDCs were collected after 9‐day induction culture, and were seeded in six‐well plates at a density of 2.0 × 10^6^ cells per well overnight. Afterward, the cells were pretreated with 2 µm dfo for 12 h, after being washed three times with PBS, cells were pulsed with 20 µg mL^−1^ OVA_257–264_ peptide (China Peptides Co., Ltd., China) for 6 h and then they were pelleted and washed with PBS and incubated with BV 421 anti‐mouse I‐A/I‐E and PE anti‐mouse CD11c Abs and APC anti‐mouse H‐2Kb Ab bound to SIINFEKL for 30 min on ice and in the dark. The cells were then washed twice with PBS and analyzed by flow cytometry.

### In Vitro Differentiation of T Cells

BMDCs (2 × 10^6^) were incubated with 20 µg mL^−1^ OVA_323–339_ peptide (China Peptides Co., Ltd., China) for 6 h and subsequently co‐cultured with spleen cells from OT‐II mice (at a 1:5 ratio) for an additional 12 h in the presence of 20 U mL^−1^ IL‐2. Following co‐culture, the cells were treated with a mixture of phorbol 12‐myristate 13‐acetate/ionomycin and brefeldin A/monensin (Hangzhou Lianke Biotechnology Co., Ltd, China) for 12 h as per the manufacturer's instructions. Subsequently, the cells were fixed, stained, and subjected to further analysis. Co‐cultured cells were initially stained with FITC‐conjugated anti‐mouse CD19, APC‐conjugated anti‐mouse CD3, PE/Cy7‐conjugated anti‐mouse CD4, and eFluor 450‐conjugated anti‐mouse CD8a Abs. They were then fixed and permeabilized using the BD Cytofix/Cytoperm solution following the manufacturer's guidelines. The fixed cells were subsequently washed with BD Perm/Wash buffer and further stained with specified Abs (BioLegend) for intracellular cytokines, including PerCP/Cy5.5‐conjugated anti‐mouse IFN‐γ Abs.

### Assessment of DC Maturation Through Flow Cytometry

DC isolation was performed using anti‐mouse CD11c Abs (eBioscience, USA) and MagniSort SAV positive selection beads (Invitrogen, USA) according to the manufacturer's instructions. Fluorescent dye‐conjugated Abs such as PE‐conjugated anti‐mouse CD40 (3/23) and PE/Cy7 conjugated anti‐mouse CD86 (GL‐1) Abs were used for surface staining and flow cytometric analysis.

### Cell Proliferation Analysis with CFSE Staining

For analysis of proliferation and expansion of T cells upon DC stimulation, CD4⁺ T cells and CD8⁺ T cells were sorted using flow cytometry (BD FACSAria III, BD, USA). These isolated CD8⁺ T cells were labeled with 5 µM CFSE at 37 °C for 10 min. After washing three times with warm PBS, the labeled cells were co‐cultured with immunomagnetic bead‐sorted DCs or BMDCs for 3 d in the presence of 20 U mL^−1^ IL‐2. After incubation, the cells were harvested, and the proliferation of CD4⁺ and CD8⁺ T cells was further detected through the analysis of generational dye dilution via flow cytometry.

### Determination of Intracellular Labile Iron Pool (LIP) in DC Progenitor Cells

DC progenitor cells were sorted by flow cytometry and 1.0 × 10⁴ cells were counted. The cells were washed twice with PBS, 500 µL of calcein‐AM (final concentration: 0.5 µm) was added, and they were incubated in the dark at 37 °C for 15 min. Cells were washed twice with PBS, resuspended in PBS, and analyzed using a flow cytometer to select the FITC channel for detection.

### RNA Sequencing

Splenocytes were isolated from mouse spleens. They were then blocked with TruStain fcX (anti‐mouse CD16/32 Abs) and incubated with CD11c‐Biotin Abs (eBioscience, USA). Subsequently, CD11c^+^ cells were isolated with MagniSort Streptavidin Positive Selection Beads (MSPB‐6003‐71, Invitrogen) according to the manufacturer's instructions.^[^ ^65^ ^]^ DC progenitor cells were stained with a FITC‐conjugated anti‐mouse lineage antibody cocktail containing APC‐conjugated anti‐mouse CD135, PE/Cy7‐conjugated anti‐mouse CD34, APC/Cy7‐conjugated anti‐mouse CD16/32, PE‐conjugated anti‐mouse CD115, and PE/Dazzle594‐conjugated anti‐mouse CD117 Abs, and then sorted using flow cytometry (BD FACSAria III, BD, USA).

Total RNA was extracted from the collected DC progenitors and CD11c^+^ cells using an RNeasy Mini Kit (Qiagen) designed for small amounts of cells. The quality and purity of total RNA were evaluated using an Agilent 2100 Bioanalyzer system (Agilent Technologies, CA, USA). RNA (2 ng) was subjected to RNA‐Seq analysis using the DNBSEQ platform (Shenzhen, China). This highly sensitive platform could analyze as little as 2 ng of total RNA.^[^ ^66^ ^]^ Each sample had an average of 6.66G of data. The average alignment rate of the samples to the genome was 93.46% and the average alignment rate to the gene set was 77.72%, indicating that the sequencing quality was high and that the sequencing depth was sufficient to cover the transcriptome.

### Validation of the Sequencing Results by Means of qRT‐PCR

Total RNA was isolated from the collected cells according to the manufacturer's instructions (AC0202‐B; Spark Jade, China). The relative expression levels of the related genes were determined using the standard ΔΔCt method. Cyclophilin A was used as the loading control for normalization. The primers used are listed in Table S1 (Supporting Information).

### Statistical Analysis

Statistical analysis was performed using the independent t‐test in SPSS software. All experimental data are shown as the mean ± standard error (SE), and *p* values (*p* < 0.05 and *p* < 0.001) define statistical significance.

## Conflict of Interest

The authors declare no conflict of interest.

## Author Contributions

Q.R., J.M., and S.L. conceptualized the study. Q.R., Z.D., J.Q., Q.S., and X.X. developed the methodology. Q.R., Z.D., J.Q., Q.S., J.M., and S.L. conducted the investigation. J.M., R.C., Y.L., and S.L. supervised the project. Q.R., J.M., and S.L. wrote the manuscript.

## Supporting information



Supporting Information

## Data Availability

The data that support the findings of this study are available in the supplementary material of this article.
