# Supplementary material for: Iron Deficiency Impairs Dendritic Cell Development and Function, Compromising Host Anti‐Infection Capacity
Source: Adv Sci (Weinh). 2025 Apr 30;12(20):2408348. doi: 10.1002/advs.202408348 (PMC12120711; doi:10.1002/advs.202408348)
Supplement: Supplementary file 1 — Supporting Information [file ADVS-12-2408348-s001.pdf]

ADVANCED SCIENCE
Open Access

Supporting Information

for *Adv. Sci.*, DOI 10.1002/advs.202408348

Iron Deficiency Impairs Dendritic Cell Development and Function, Compromising Host Anti-Infection Capacity

Quanzhong Ren, Xiaotong Xu, Zheng Dong, Jiahuang Qiu, Qing'e Shan, Rui Chen, Yajun Liu, Juan Ma and Sijin Liu*

Supporting Information for

Iron deficiency Impairs Dendritic Cell Development and Function, Compromising Host's Anti-Infection Capacity

Quanzhong Ren, Xiaotong Xu, Zheng Dong, Jiahuang Qiu, Qing'e Shan, Rui Chen, Yajun Liu, Juan Ma, Sijin Liu*

Q. Ren, J. Qiu, X. Xu, J. Ma, S. Liu
State Key Laboratory of Environmental Chemistry and Ecotoxicology, Research Center for Eco-Environmental Sciences, Chinese Academy of Sciences, Beijing 100085, P.R. China.
Q. Ren, Y. Liu
JST sarcopenia Research Centre, National Center for Orthopaedics, Beijing Research Institute of Traumatology and Orthopaedics, Beijing Jishuitan Hospital, Capital Medical University, Beijing, 100035, China.
R. Chen
Department of Toxicology and Sanitary Chemistry, School of Public Health, Capital Medical University, Beijing, 100069, P.R. China.
J. Qiu, X. Xu, J. Ma, S. Liu
University of Chinese Academy of Sciences, Beijing,100049, P.R. China.
Z. Dong, Q. Shan, S. Liu
Medical Science and Technology Innovation Center, Shandong First Medical University & Shandong Academy of Medical Sciences, Jinan, Shandong, 250117, P.R. China.
*Corresponding authors
Email: juanm@rcees.ac.cn

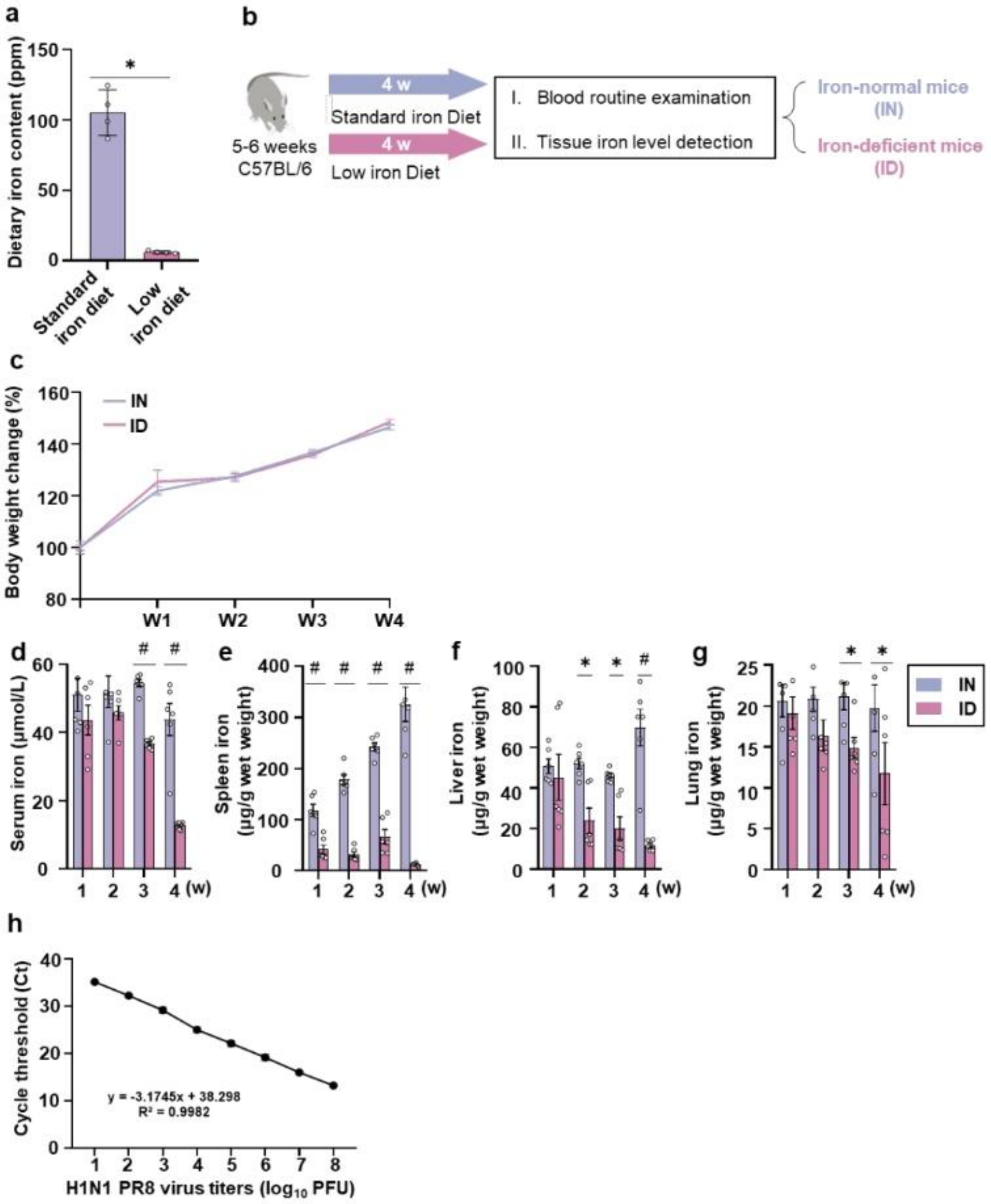

Supplementary Fig. 1. Effect of the low-iron diet on body weight iron levels in serum and tissues, and H1N1 PR8 viral titers. (a) Inductively coupled plasma-mass spectrometry (ICP-MS) was used to measure iron content of the diets. (b) A schematic diagram illustrating the establishment of the ID mice model. (c) Weekly body weight of mice fed a low-iron diet administration for 4 weeks (n = 5). (d–g) The iron content of serum (d), spleen (e), liver (f) and lung (g) from IN and ID mice after 4 weeks of a low iron diet (n = 6). (h) The standard curve of viral titers was generated by analyzing the viral matrix protein 1 (M1) gene using SYBR Green-based qRT-PCR, and it was created from 10-fold serial dilutions of the virus stocks (n = 6). The coefficient of determination (R^2) and the slope of the regression curve are shown. $P < 0.05$ (*) and $P < 0.001$ (#).

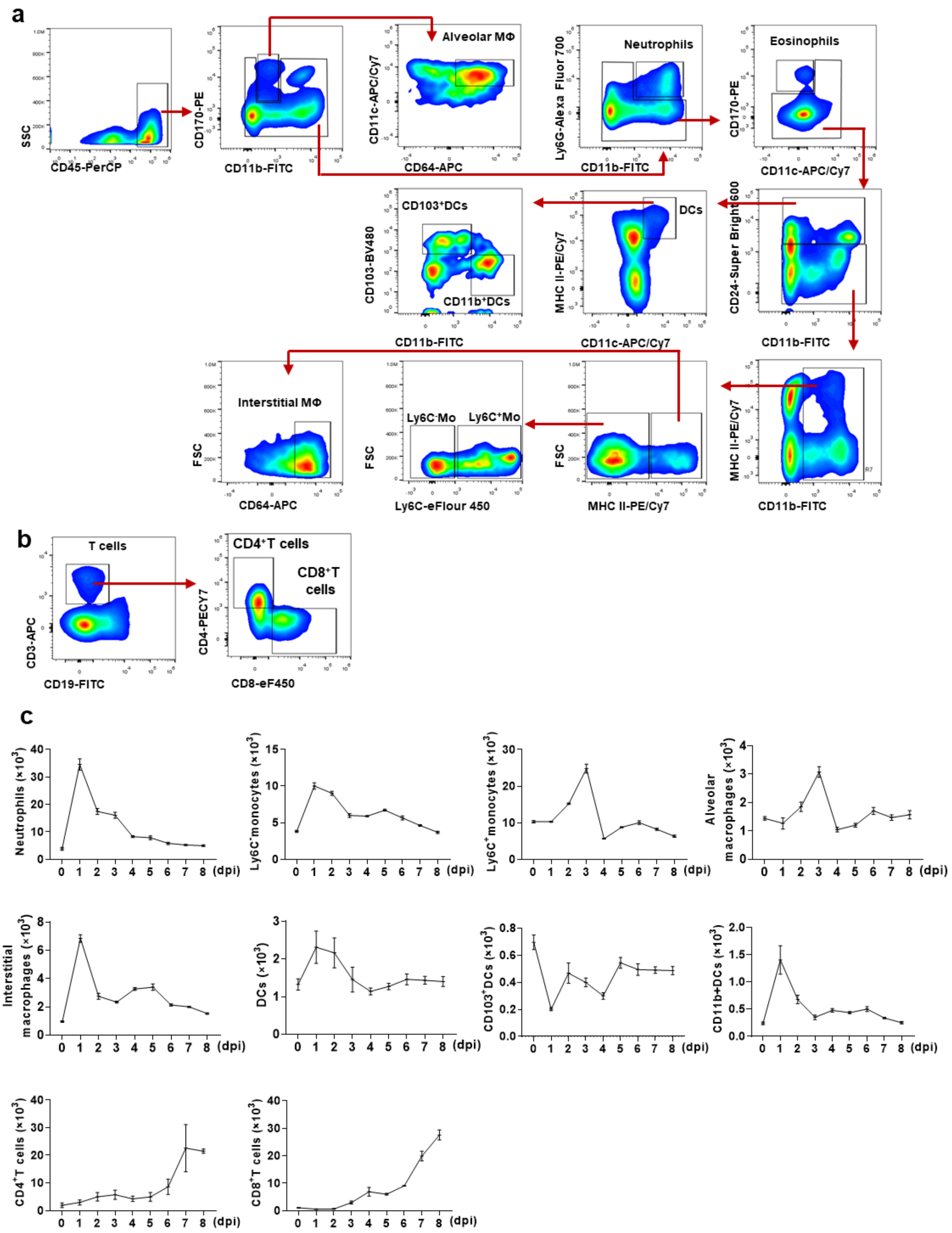

Supplementary Fig. 2. Changes in pulmonary immune cells at different time points after influenza virus infection. (a, b) The gating strategies in flow cytometry for the analysis of (a) innate immune cells and (b) adaptive immune cells in lung single-cell suspension. (c) Flow

cytometry analysis of the changes of immune cells from lung single-cell suspensions obtained from infected and uninfected mice from day 0 to day 8, respectively (n = 5).

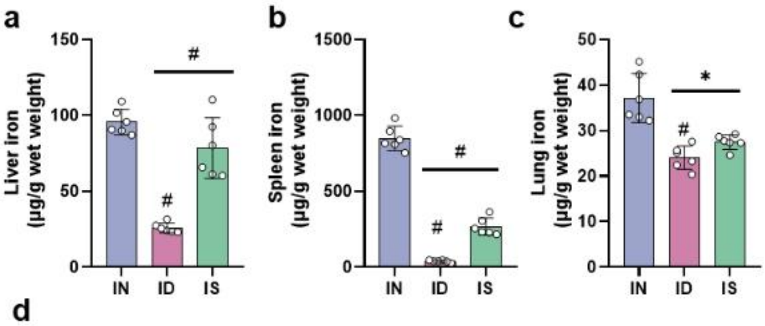

d

Group	IN	ID	IS
RBC (10^{12}/L)	8.71±0.33	8.85±0.09	8.86±0.2
HGB (g/L)	126.2±5.12	**116.8±1.79***	125.2±3.42
HCT (%)	40.3±1.64	38.96±0.5	41.34±1.44

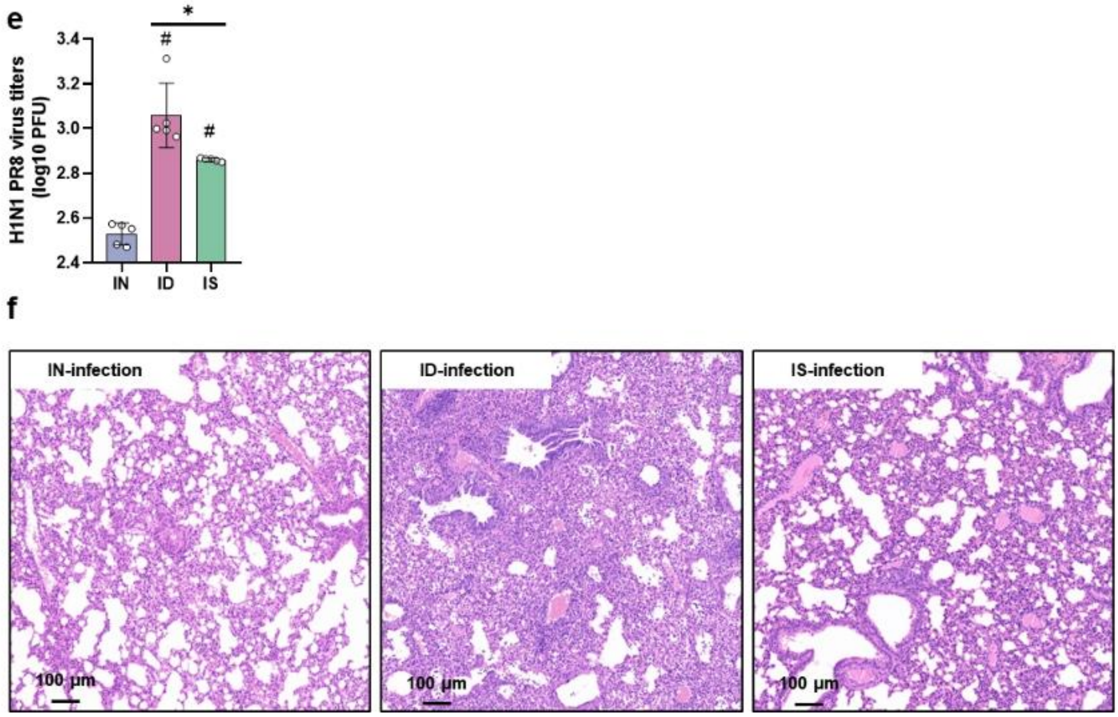

Supplementary Fig. 3. The antiviral ability of ID mice is restored after dietary iron supplementation. (a-c) The iron content in the (a) liver, (b) spleen, and (c) lung of IN, ID and IS mice (n = 6). (d) Changes in red blood cell count (RBC, 10^{12}/L), hemoglobin concentration (HGB, g/L), and hematocrit (HCT, %) of IN, ID and IS mice (n = 5). (e) qRT-PCR analysis of viral titers in lungs from mice 7 d after virus infection (n = 3). (f) Representative H&E stained lung sections of IN, ID and IS mice 2 weeks after treatment with or without virus. $P < 0.05$ (*) and $P < 0.001$ (#) for comparisons as indicated by the horizontal bars, or relative to IN mice. IS: iron-supplemented.

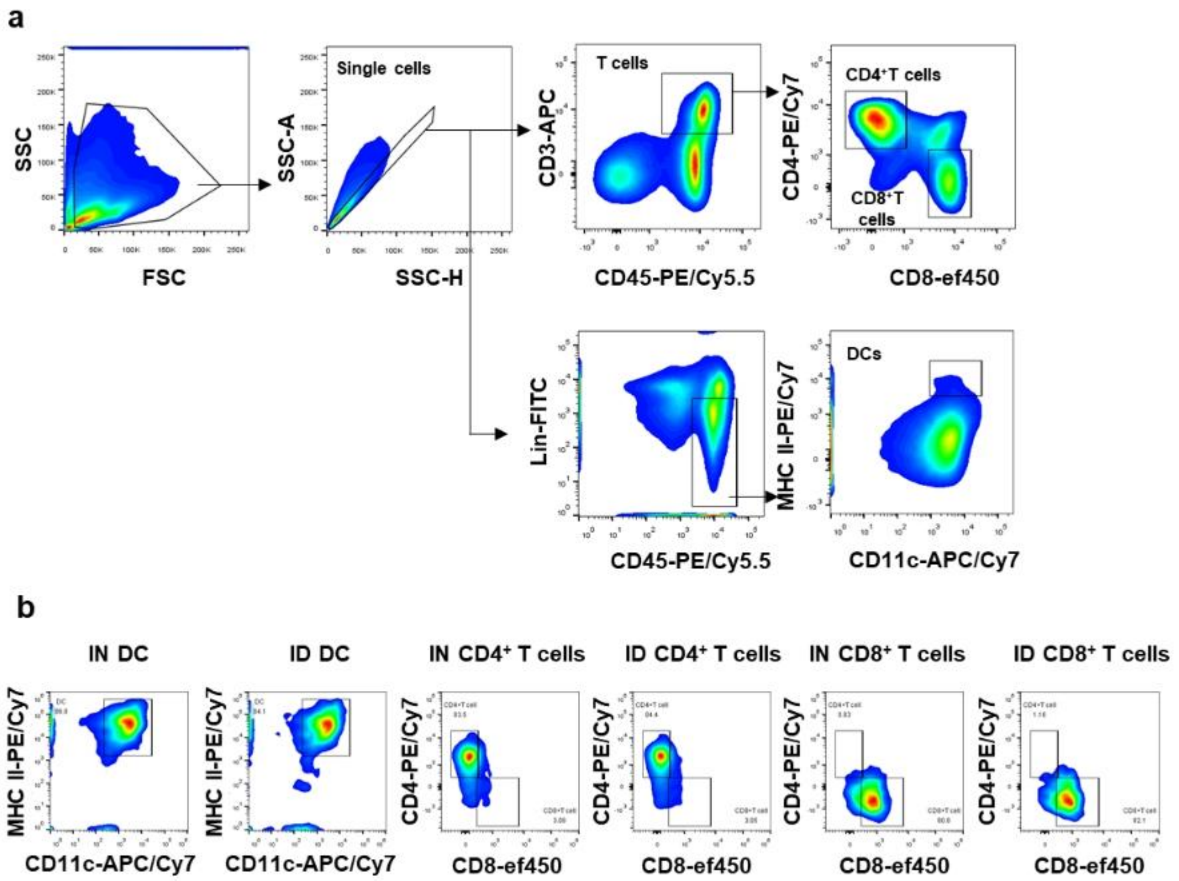

Supplementary Fig. 4. Sorting and verification of the purity of DCs, CD4^{+} T cells, and CD8^{+} T cells. (a) Flow cytometry was used to sort CD4^{+} T cells, CD8^{+} T cells and DCs in the lungs after influenza virus infection. (b) The purity of the collected cells was verified by flow cytometry.

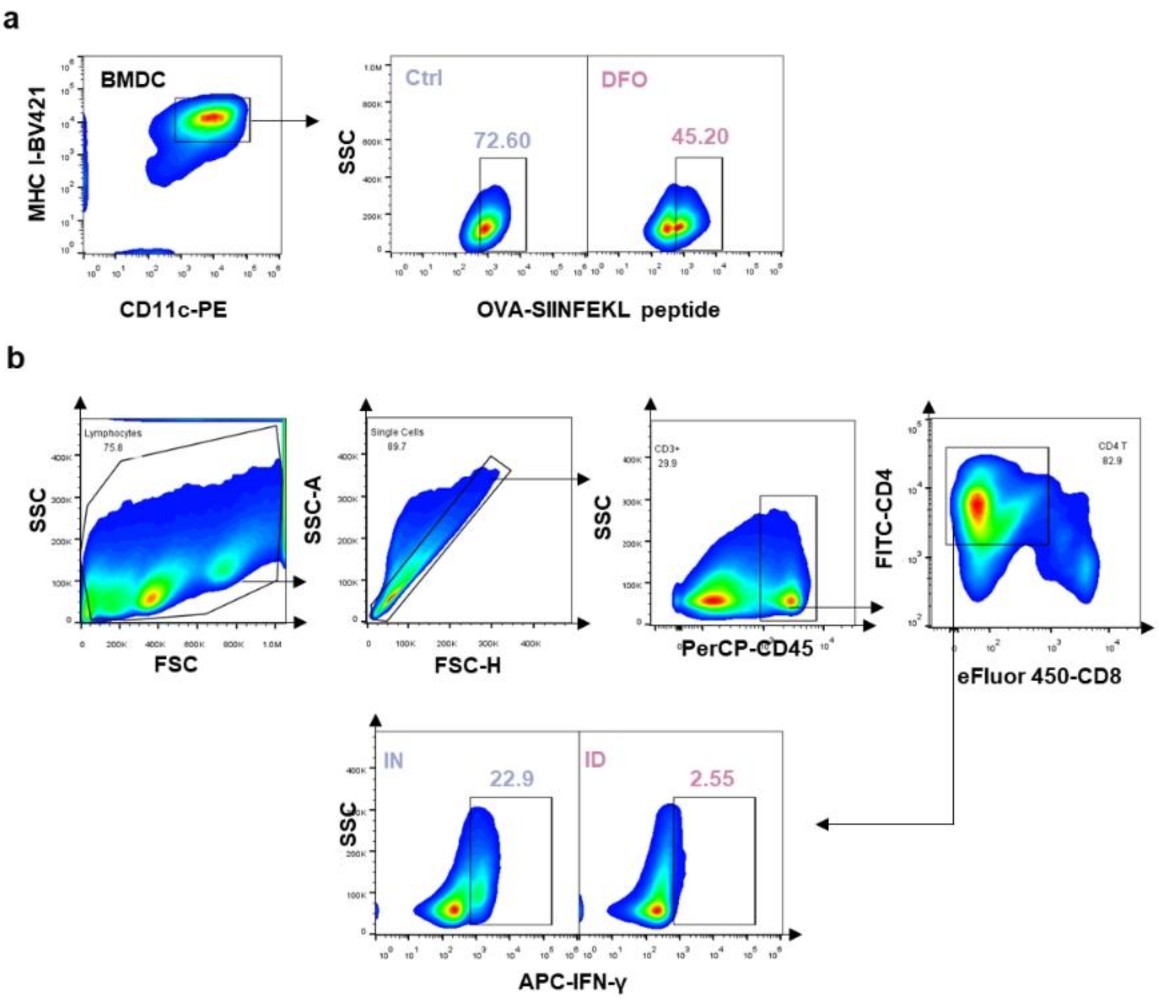

Supplementary Fig. 5. ***An in vitro*** **test was conducted to examine the decreased antigen presentation ability of ID mouse-derived DCs.** (a) Flow cytometry analysis of the percentage of H2Kb SIINFEKL^{+} cells after treating bone-marrow-derived cells with or without desferrioxamine (2 μM) for 24 h. (b) Flow cytometry analysis of the level of IFN-γ secreted by splenic cells stimulated by DCs derived from the spleens of IN and ID mice.

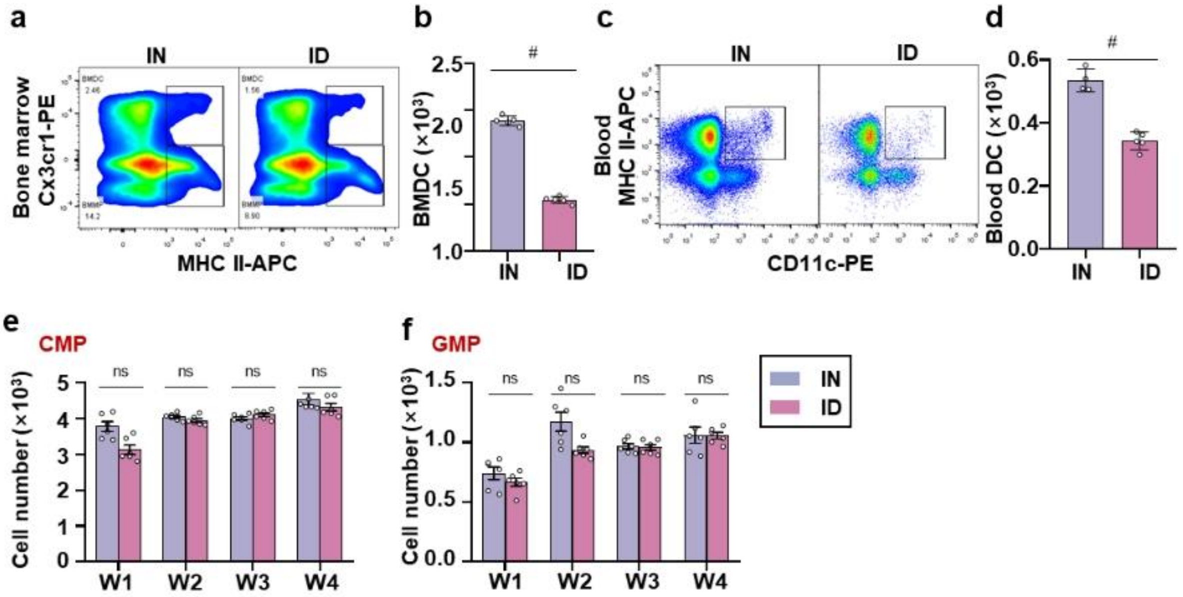

Supplementary Fig. 6. DC numbers in ID mice were significantly reduced in both bone marrow and peripheral blood. (a–d) Flow cytometry analysis of the number of DCs in **(**a, b) bone marrow and (c, d) blood derived from IN and ID groups, respectively (n = 5). The IN mice received a normal iron diet only. *P* < 0.001 (#) relative to the IN mice. **(**e, f) Flow cytometry analysis of the number of common myeloid progenitor (CMP, $Lin^{-}CD117^{+}CD16/32^{Lo}CD34^{+}$, e) and granulocyte–macrophage progenitor (GMP, $Lin^{-}CD117^{+}CD16/32^{Lo}CD34^{-}$, f) cells derived from the IN and ID groups, respectively (n = 5).

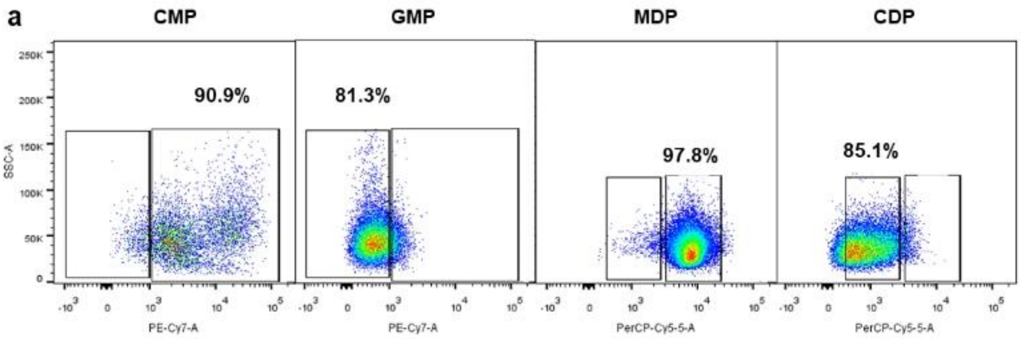

Supplementary Fig. 7. Flow cytometry was used to validate the purity of sorted DC progenitor cells.

Supplementary Table 1. Primers sequences used in this study.

Genes	Sequences (5'-3')
PR8-M1-forward	AAGACCAATCCTGTCACCTCTGA
PR8-M1- reverse	CAAAGCGTCTACGCTGCAGTCC
Tlr2-forward	GCAAACGCTGTTCTGCTCAG
Tlr2- reverse	AGGCGTCTCCCTCTATTGTATT
Tlr4-forward	ATGGCATGGCTTACACCACC
Tlr4- reverse	GAGGCCAATTTTGTCTCCACA
Mmp9-forward	CTGGACAGCCAGACACTAAAG
Mmp9- reverse	CTCGCGGCAAGTCTTCAGAG
Il6-forward	TAGTCCTTCCTACCCCAATTTCC
Il6- reverse	TTGGTCCTTAGCCACTCCTTC
Bcl3-forward	CCGGAGGCCCTTTACTACCA
Bcl3- reverse	GGAGTAGGGGTGAGTAGGCAG
Bax-forward	TGAAGACAGGGGCCTTTTTG
Bax- reverse	AATTCGCCGGAGACACTCG
Jun-forward	CCTTCTACGACGATGCCCTC
Jun- reverse	GGTTCAAGGTCATGCTCTGTTT
Cyclophilin A-forward	AAGAAGGCATGAACATTGTGGAAGC
Cyclophilin A-reverse	CGGAAATGGTGATCTTCTTGCTGG

Supplementary Table 2. Detailed information of fluorescent dye-conjugated Abs used in flow cytometry.

Anti-mouse Ab	Fluorescent dye	Clone	Resource
CD45	PerCP	30-F11	Biolegend Inc.
CD11b	FITC	M1/70	Biolegend Inc.
I-A/I-E	PE/Cy7	M5/114.15.2	Biolegend Inc.
CD170	PE	S17007L	Biolegend Inc.
CD11c	APC/Cy7	N418	Biolegend Inc.
CD64	APC	S18017D	Biolegend Inc.
CD24	BV605™	M1/69	Biolegend Inc.
Ly-6G	BV711	1A8	Biolegend Inc.
Ly-6C	BV510	HK1.4	Biolegend Inc.
F4/80	PE	BM8	Biolegend Inc.
CD11c	PE	N418	Biolegend Inc.
I-A/I-E	APC	M5/114.15.2	Biolegend Inc.
I-A/I-E	BV 421	M5/114.15.2	Biolegend Inc.
CD11b	BV 605	M1/70	Biolegend Inc.
CD135	APC	A2F10	Biolegend Inc.
CD34	PE/Cy7	HM34	Biolegend Inc.
CD16/32	APC/Cy7	S17011E	Biolegend Inc.
CD115	PE	AFS98	Biolegend Inc.
CD19	FITC	1D3/CD19	Biolegend Inc.
CD3	APC	17A2	Biolegend Inc.
CD4	PE/Cy7	GK1.5	Biolegend Inc.
IFN-γ	Percp/cy5.5	XMG1.2	Biolegend Inc.
IL-4	PE	11B11	Biolegend Inc.
CD25	BV510	PC61	Biolegend Inc.
FOXP3	Alexa Fluor® 700	MF-14	Biolegend Inc.
CD40	PE	3/23	Biolegend Inc.
CD86	PE/Cy7	GL-1	Biolegend Inc.
H-2Kb bound to SIINFEKL	APC	25-D1.16	Biolegend Inc.
CD8a	eFluor™ 450	53-6.7	Thermo Fisher Scientific. Inc.
CD135	APC	A2F10	Thermo Fisher Scientific. Inc.
CD117	PE-eFluor™ 610	2B8	Thermo Fisher Scientific. Inc.
CD34	eFluor 450	RAM34	Thermo Fisher Scientific. Inc.
CD16/CD32	PE-Cyanine7	93	Thermo Fisher Scientific. Inc.
CD24	Super Bright™ 600	M1/69	Thermo Fisher Scientific. Inc.
l-A/1-E	PE-Cyanine7	M5/114.15.2	Thermo Fisher Scientific. Inc.

Anti-mouse Ab	Fluorescent dye	Clone	Resource
Ly-6C	eFluor 450	HK1.4	Thermo Fisher Scientific. Inc.
CD103	Brilliant Violet™ 480	2E7	Thermo Fisher Scientific. Inc.
Lineage Antibody Cocktail	FITC	17A2, RA3-6B2, M1/70, TER-119, RB6-8C5	Thermo Fisher Scientific. Inc.
